# The Role of Glutamine in Growth, Health and Sensory Quality of Female Rainbow Trout (*Oncorhynchus mykiss*)

**DOI:** 10.1002/vms3.70527

**Published:** 2025-07-28

**Authors:** Mustafa Öz, Burak Evren İnanan

**Affiliations:** ^1^ Department of Fisheries and Diseases, Faculty of Veterinary Medicine Aksaray University Aksaray Turkey

**Keywords:** antioxidant capacity, functional feed additive, glutamine, rainbow trout, sensory evaluation

## Abstract

**Objectives:**

This study aimed to investigate the effects of dietary glutamine supplementation on growth performance, haematological and biochemical responses, oxidative stress parameters and sensory quality of fillets in female rainbow trout.

**Methodology:**

A total of 360 female rainbow trout were randomly divided into four groups and fed diets supplemented with 0.0% (control), 0.5%, 1.5% and 2.0% glutamine for 60 days. Growth indices were measured, and blood samples were analysed for haematological and biochemical parameters. Muscle tissue was examined for oxidative stress markers. Sensory evaluation was performed on fillets by trained panellist. Data were analysed using one‐way ANOVA and Duncan's multiple comparison test.

**Results:**

Glutamine supplementation at 1.5% and 2.0% significantly improved final body weight, weight gain and feed conversion ratio (*p* < 0.05). Haematological indices, such as red blood cell (RBC), haemoglobin and haematocrit levels, increased in the treated groups. Significant enhancements in total protein and albumin levels were observed, along with reduced malondialdehyde (MDA) levels, indicating improved oxidative status. Sensory evaluation revealed better odour, flavour and texture in fillets from glutamine‐supplemented groups, particularly at 1.5% and 2.0% inclusion levels.

**Conclusions:**

Dietary glutamine supplementation, particularly at 1.5%–2.0%, enhances growth, physiological health, oxidative balance and sensory characteristics in female rainbow trout. These findings support the practical use of glutamine as a functional feed additive in sustainable aquaculture systems.

## Graphical abstract

Dietary supplementation with 1.5%–2.0% glutamine significantly improved growth performance, feed efficiency and protein deposition while reducing lipid accumulation in female rainbow trout (*Oncorhynchus mykiss*). Glutamine also enhanced antioxidant capacity (↑ TAS, ↓ MDA) and improved sensory attributes of cooked fillets, particularly texture and flavour. These findings support the use of glutamine as an effective functional feed additive in trout aquaculture.

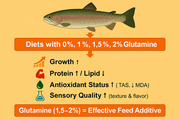

## 1 Introduction

The aquaculture sector has expanded significantly over recent decades, with rainbow trout (*Oncorhynchus mykiss*) emerging as a species of both ecological adaptability and economic value. Originally cultivated for regional markets, rainbow trout farming has grown into a global enterprise. Global production increased from 180000 t in 1980 to 773000 t in 2022 (FAO 2025), with Türkiye contributing approximately 25% of this volume. As demand continues to rise, the industry has shifted toward producing larger trout, typically exceeding 1 kg, which are now exported under the name of Turkish salmon. Among the cultured stocks, female trout are especially favoured due to their higher fillet yield, healthier lipid composition and better muscle quality compared to males.

In addition to these market‐driven preferences, biological factors such as sex also play a significant role in determining growth performance and meat quality. Female rainbow trout have been shown to outperform males in several physiological and product‐related metrics, including fillet weight and nutritional profile (Aussanasuwannakul et al. 2011; Manor et al. 2015). However, realizing the full potential of these advantages requires feeding strategies that are specifically tailored to their metabolic needs.

In areas where carnivorous species like rainbow trout are extensively cultivated, the development of effective diets is crucial for the sustainable growth of the aquaculture industry. Functional feed additives are garnering significant interest for their capacity to enhance nutrient consumption, immunological competence and oxidative balance in aquaculture (Öz 2025). Among these, amino acids with conditionally essential roles such as glutamine and taurine are of particular importance due to their involvement in protein turnover, immune regulation and stress adaptation (Han et al. 2014; Li et al. 2020).

Glutamine is generally classified as a non‐essential amino acid, but under stress or high metabolic demand, it becomes essential. It serves multiple physiological roles, including nitrogen transport, protein synthesis and maintaining intestinal health. In aquatic species, dietary glutamine has been linked to improved growth rates, antioxidant capacity and immune responses (Hu et al. 2015; Huang et al. 2024). Moreover, it contributes to gut integrity and may enhance nutrient absorption and overall health (Li et al. 2020). Its functions also extend to ammonia detoxification and nitrogen homeostasis. For instance, Wicks and Randall (2002) observed elevated glutamine concentrations in the brain and liver of rainbow trout, suggesting a buffering role against environmental and dietary ammonia exposure.

During reproductive maturation, female rainbow trout often undergo muscle protein catabolism, which negatively affects fillet structure and protein content (Yamashita and Konagaya 1990). In such cases, feed additives that preserve muscle quality while supporting growth could offer significant benefits. Although the growth‐promoting effects of glutamine have been reported in various studies (Öz et al. 2024a), its influence on sensory traits, particularly in female fish, has not been thoroughly explored.

This study aims to evaluate the effects of dietary glutamine supplementation on the growth performance, haematological parameters, oxidative stress markers and fillet quality of female rainbow trout. Particular attention is given to the nutritional and sensory properties of the muscle tissue, with the overall goal of assessing the potential of glutamine as a functional feed additive for sustainable trout aquaculture.

## 2 Materials and Methods

### 2.1 Preparation of the Fish and Diet

The experimental procedures were approved by the Local Animal Ethics Committee in Adana, Türkiye (Approval No. 9‐1/2021). Prior to the trial, rainbow trout (*O. mykiss*) were subjected to a 14‐day acclimation period, during which they were fed a commercial diet (Skretting, Stavanger, Norway) containing 43% crude protein, 24% crude lipid, 3.9% crude fibre and 9% ash. This basal diet was also used as the control.

Following acclimation, 360 female trout with an average initial weight of 412.58 ± 8.76 g were randomly distributed into 12 floating cages (3 m^3^ each, 30 fish per cage). Four dietary treatments were prepared by supplementing the basal diet with l‐glutamine at inclusion levels of 0.0% (G1 as control), 1.0% (G2), 1.5% (G3) and 2.0% (G4), based on prior findings (Öz, Inanan et al. 2024a). Glutamine powder was first dissolved in 500 mL of water and uniformly sprayed onto the feed. Sunflower oil was then added to minimize leaching. The coated feed was air‐dried in the shade and stored in sealed containers until use.

Fish were hand‐fed twice daily to apparent satiation for 165 days. Water temperature ranged between 10°C and 16°C, and dissolved oxygen levels were maintained above 9 mg/L throughout the trial.

### 2.2 Calculations of Growth‐Related Performance

The following formulae was used in calculation of feed utilization and growth performance: specific growth rate (SGR, % day^−1^) = 100 × (ln *w*_1_ − ln *w*_0_)/*t*, where *w*_1_ and *w*_0_ are the wet weights at times *t*_1_ and *t*_0_ (Company et al. 1999).

The feed conversion ratio (FCR) = consumed feed/(*W*_final_ − *W*_initial_), where *W*_final_ and *W*_initial_ are the live weights (g) of the fish on the initial (*t*) and final (*T*) days, respectively (Santinha et al. 1999).

The protein efficiency ratio (PER) = weight gain (g)/protein intake (g) (Skalli et al. 2004).

### 2.3 Blood Sampling and Analysis

At the conclusion of the feeding trial, fish were anaesthetized with 300 ppm of 2‐phenoxyethanol and surface‐disinfected with 70% ethanol before blood collection. Blood samples were drawn from the vena caudalis using heparinized syringes (Öz, Üstüner et al. 2024b; Öz et al. 2024c):

- Haematological analysis: Blood samples designated for haematological evaluation were transferred into lavender‐top tubes containing EDTA.
- Serum biochemical analysis: The remaining blood was placed in red‐top serum separator tubes (SST II) and centrifuged at 13,000 × *g* for 10 min at 4°C to separate serum. The obtained serum was immediately analysed or stored at −80°C for future biochemical assessments.

Six fish (*n* = 6) per group were used for haematological and serum biochemical analyses.

Haematological parameters, including red blood cell count (RBC), mean cell volume (MCV), mean cell haemoglobin (MCH), mean cell haemoglobin concentration (MCHC), haematocrit (Hct) and haemoglobin (Hb), were assessed using an automated haematology analyser (MS4‐S, Melet Schloesing Laboratories, Osny, France). To validate the automated readings, a manual haematological assessment was performed following the methodology of Blaxhall and Daisley (1973).

Serum biochemical parameters, such as alkaline phosphatase (ALP), aspartate aminotransferase (AST), alanine aminotransferase (ALT), glucose (GLU), albumin (ALB), cholesterol (CHOL), total protein (TP) and globulin (GLO), were analysed using the MScan II automated biochemical analyser (Melet Schloesing, Osny, France) in accordance with the manufacturer's protocols.

### 2.4 Oxidative Stress Parameters

Oxidative stress biomarkers were evaluated using blood and muscle samples collected from six fish (*n* = 6) per treatment group. The evaluation of oxidative stress indicators was conducted as follows:

- Total antioxidant status (TAS) was determined using Relassay commercial kits (Cat. No.: RL0017, Turkey), based on the method described by Erel (2004).
- Total oxidant status (TOS) was measured using Relassay kits (Cat. No.: RL0024, Turkey), following Erel (2005).
- The oxidative stress index (OSI) was calculated as follows: OSI (arbitrary units) = TOS (µmol H_2_O_2_ equivalent/L)/TAS (µmol Trolox equivalents/L) (Yumru et al. 2009; Kosecik et al. 2005; Harma et al. 2003).
- Malondialdehyde (MDA) levels, a key lipid peroxidation marker, were analysed on the basis of the protocol described by Alak et al. (2020).
- Myeloperoxidase (MPO) enzyme activity was determined **separately** in erythrocyte and muscle tissue samples following the methodology of Bradley et al. (1993), in order to assess tissue‐specific responses.
- Total protein concentrations in samples were measured spectrophotometrically at 595 nm, using the Bradford method (1976).

### 2.5 Sensory Analysis

Sensory attributes of raw and cooked fillets were assessed following the quality index method (QIM) scheme, originally developed for fresh cod (*Gadus morhua*) by Bonilla et al. (2007), with slight modifications. The QIM included 11 quality parameters, such as

- Skin characteristics: Brightness, mucus and scale integrity.
- Flesh properties: Texture, blood presence, odour, colour, brightness, aroma and gaping.
- Eye attributes: Visibility, shape and iris clarity.

Each parameter was scored from 0 to 3, where 0 represented the best quality and higher scores indicated lower quality, with a maximum demerit score of 20.

For cooked fillets, sensory evaluation followed Paulus et al. (1979). Samples were prepared using a microwave oven (450 W, 3 min) and assessed for colour, odour, flavour, texture and overall acceptability on a 9‐point hedonic scale (1 = dislike extremely, 9 = like extremely).

Six formally trained panellists performed blind assessments, and palates were neutralized with water between tastings.

### 2.6 Proximate Composition of Fish Muscle

The chemical composition of fish fillets was determined using standard methodologies:

- Lipid content: Analysed using Bligh and Dyer's method (1959).
- Moisture and ash content: Assessed following AOAC (1990) protocols.
- Crude protein content: Measured using the Kjeldahl method (AOAC 1984).

### 2.7 Statistics

Statistical evaluations were conducted using SPSS version 18.0 (IBM Corp., USA). To compare the experimental groups, one‐way ANOVA was employed. Upon detection of statistically significant differences (*p* < 0.05), Duncan's multiple range test was used to identify group‐wise variations. All findings are expressed as mean values accompanied by standard deviations (±SD).

## 3 Results

### 3.1 Growth Performance of Rainbow Trout

Table 1 presents the growth performance outcomes for female rainbow trout subjected to dietary glutamine supplementation. Individuals receiving glutamine‐enriched diets (G2, G3 and G4) exhibited statistically significant improvements in final body weight (FW), weight gain (WG), SGR and PER compared to the control group (G1) (*p* < 0.05). The highest values for FW (1397.11 ± 6.76 g), WG (984.53 ± 6.76 g) and SGR (0.63 ± 0.01) were recorded in the G4 group, which received 2.0% dietary glutamine. Moreover, FCR was markedly lower in the G3 and G4 groups (1.11 ± 0.01), suggesting enhanced feed utilization at elevated glutamine levels.

**TABLE 1:** Growth performance of rainbow trout fed with diets supplemented with different ratios of glutamine.

	G1‐control	G2‐1.0%	G3‐1.5%	G4‐2.0%
**IW**	412.58 ± 8.76	412.58 ± 8.76	412.58 ± 8.76	412.58 ± 8.76
**FW**	1129.69 ± 5.55^d^	1257.35 ± 7.49^c^	1378.48 ± 8.66^b^	1397.11 ± 6.76^a^
**WG**	717.11 ± 5.55^d^	844.77 ± 7.49^c^	965.90 ± 8.67^b^	984.53 ± 6.76^a^
**FCR**	1.48 ± 0.02^a^	1.31 ± 0.01^b^	1.11 ± 0.01^c^	1.11 ± 0.01^c^
**SGR**	0.51 ± 0.01^d^	0.55 ± 0.00^c^	0.60 ± 0.01^b^	0.63 ± 0.01^a^
**PER**	1.57 ± 0.02^c^	1.78 ± 0.01^b^	2.08 ± 0.03^a^	2.09 ± 0.02^a^
**FI**	1064.58 ± 5.12^c^	1103.99 ± 5.96^a^	1077.53 ± 7.25^b^	1093.17 ± 8.56^a^

*Note*: The averages which are expressed using diferent letters in each row are signifcantly diferent (*p* < 0.05)

Abbreviations: FCR, feed conversion rates; FI, feed intake; FW, final weight; IW, initial weight; PER, protein efficiency ratio; SGR, specific growth rate.

Similarly, PER increased significantly with glutamine inclusion, with the highest values recorded in the G3 (2.08 ± 0.03) and G4 (2.09 ± 0.02) groups (*p* < 0.05). These results suggest enhanced protein utilization in glutamine‐fed fish.

Although feed intake (FI) did not differ significantly among treatments, a slight increase was noted in the G2 and G4 groups, which may imply a mild stimulatory effect of glutamine on feeding behaviour.

Collectively, the data indicate that glutamine supplementation improved growth performance by promoting protein assimilation and feed utilization in female rainbow trout.

### 3.2 Muscle Nutrient Content

The results of muscle nutrient composition analysis (Table 2) indicate that dietary glutamine supplementation significantly influenced the crude protein and lipid content of female rainbow trout (*p* < 0.05). Crude protein levels increased with higher glutamine inclusion, reaching the highest value in the G4 group (20.47% ± 0.08%), whereas total lipid content decreased significantly, with the lowest value observed in the G4 group (3.04% ± 0.12%). Additionally, ash content increased, whereas moisture content slightly decreased in glutamine‐fed groups (*p* < 0.05).

**TABLE 2:** Effects of dietary glutamine inclusion on the proximate composition of rainbow trout muscle after 165 days of feeding.

Traits (%)	G1‐control	G2‐1.0%	G3‐1.5%	G4‐2.0%
**Crude protein**	17.67 ± 0.09^d^	18.56 ± 0.20^c^	19.98 ± 0.19^b^	20.47 ± 0.08^a^
**Total lipid**	4.71 ± 0.10^a^	3.76 ± 0.15^b^	3.29 ± 0.06^c^	3.04 ± 0.12^d^
**Ash**	1.92 ± 0.04^d^	2.05 ± 0.04^c^	2.32 ± 0.05^b^	2.44 ± 0.05^a^
**Moisture**	75.33 ± 0.16^d^	75.09 ± 0.09^c^	73.98 ± 0.22^b^	73.58 ± 0.19^a^

*Note*: The averages which are expressed using diferent letters in each row are signifcantly diferent (*p* < 0.05)

These findings suggest that glutamine supplementation improves muscle protein deposition while reducing lipid accumulation, contributing to enhanced fillet quality.

### 3.3 Raw and Cooked Sensory Evaluations

In the sensory evaluation of both raw and cooked rainbow trout, the fish were fed with glutamine‐supplemented feed at varying rates. For the raw sensory evaluation, expert panellists assessed the fish based on skin (shine, mucus and scales), eyes (prominence, eye shape and iris) and muscle texture (texture, hardness, blood, odour and aroma). No significant differences were observed between the groups for any of these raw attributes (*p* < 0.05). Regarding the cooked sensory evaluation, fish were rated according to colour, odour, flavour, texture structure and general acceptability. The addition of glutamine to the fish feed positively influenced the texture structure and flavour but had no effect on colour or odour. These cooked sensory analysis results are summarized in Table 3. These findings indicate that although glutamine supplementation did not significantly impact on the raw sensory characteristics or affect the colour and mucus of cooked trout, it improved both the flavour and texture structure of the cooked product.

**TABLE 3:** Sensory evaluation of cooked female rainbow trout fed diets with varying levels of glutamine supplementation.

Parameters	G1‐control	G2 (1.0%)	G3 (1.5%)	G4 (2.0%)
**Colour**	8.83 ± 0.41^a^	8.83 ± 0.41^a^	9.00 ± 0.00^a^	9.00 ± 0.00^a^
**Odour**	8.83 ± 0.41^a^	9.00 ± 0.00^a^	9.00 ± 0.00^a^	9.00 ± 0.00^a^
**Flavour**	7.83 ± 0.41^b^	8.67 ± 0.51^a^	9.00 ± 0.00^a^	9.00 ± 0.00^a^
**Texture structure**	7.83 ± 0.41^b^	8.83 ± 0.41^a^	9.00 ± 0.00^a^	9.00 ± 0.00^a^
**General acceptability**	8.67 ± 0.52^a^	8.83 ± 0.41^a^	9.00 ± 0.00^a^	9.00 ± 0.00^a^

*Note*: ±standard deviation, *n* = 6 panellists. There is a statistical difference between those shown with different letters (a,b) in the same line (*p* < 0.05). Group 1: 0.0% glutamine in fish feed; Group 2: 1.0% glutamine in fish feed; Group 3: 1.5% glutamine in fish feed and Group 4: 2.0% glutamine in fish feed.

### 3.4 Effects of Glutamine on Blood Parameters

The results of blood parameter analysis (Table 4) demonstrate that dietary glutamine supplementation significantly influenced haematological indices in female rainbow trout (*p* < 0.05). RBC count, Hb and Hct levels increased with higher glutamine inclusion, with the highest values observed in the G4 group (1.88 ± 0.03 × 10^6^/µL, 15.68 ± 0.36 g/dL and 38.32% ± 0.35%, respectively). These improvements indicate enhanced oxygen‐carrying capacity and overall better physiological status in fish fed glutamine‐supplemented diets.

**TABLE 4:** Blood parameters of rainbow trout fed with different ratios of glutamine for 165 days.

	G1‐control	G2‐1.0%	G3‐1.5%	G4‐2.0%
**RBC (m/mm^3^)**	1.65 ± 0.05^c^	1.82 ± 0.04^b^	1.85 ± 0.03^ab^	1.88 ± 0.03^a^
**Hb (g/dL)**	11.30 ± 0.05^d^	13.01 ± 0.21^c^	14.80 ± 0.45^b^	15.68 ± 0.36^a^
**Hct (%)**	35.30 ± 0.34^c^	37.02 ± 0.32^b^	38.05 ± 0.23^a^	38.32 ± 0.35^a^
**MCV (fL)**	213.68 ± 7.28^a^	203.11 ± 5.79^b^	205.71 ± 2.56^b^	203.68 ± 4.44^b^
**MCH (pg)**	68.36 ± 1.83^d^	71.41 ± 1.81^c^	80.01 ± 2.65^b^	83.37 ± 2.59^a^
**MCHC (g/dL)**	32.00 ± 0.35^d^	35.17 ± 0.59^c^	38.89 ± 1.14^b^	40.94 ± 1.13^a^

*Note*: There is a statistical difference between the data shown with different letters (a,b) in the same row (*p* < 0.05). RBC, erythrocyte.

Abbreviations: Hb, haemoglobin; Hct, haematocrit; MCH, mean red blood cell haemoglobin; MCHC, mean red blood cell haemoglobin concentration; MCV, mean red blood cell volume; RBC, red blood cell.

MCV and MCHC values exhibited significant variations among groups (*p* < 0.05). MCHC increased progressively with higher glutamine levels, reaching its highest value in the G4 group (40.94 ± 1.13 g/dL), whereas MCV values slightly decreased in supplemented groups.

These findings suggest that dietary glutamine supplementation positively affects the haematological profile of female rainbow trout, potentially improving their overall health and physiological resilience.

### 3.5 Blood Serum Biochemistry Parameters of Rainbow

Serum biochemical analysis revealed that dietary glutamine supplementation significantly altered several blood parameters in female rainbow trout (Table 5). Total protein (TP) levels increased in a dose‐dependent manner, with the highest concentration detected in the G4 group (3.78 ± 0.02 g/dL), suggesting enhanced protein metabolism and systemic physiological condition (*p* < 0.05).

**TABLE 5:** Blood serum biochemistry parameters of rainbow trout fed with different ratios of glutamine for 165 days.

	G1‐control	G2‐1.0%	G3‐1.5%	G4‐2.0%
**ALP (U/L)**	127.53 ± 1.56^a^	124.10 ± 1.10^b^	119.54 ± 1.54^c^	117.33 ± 0.12^c^
**AST (U/L)**	419.99 ± 1.28^a^	403.79 ± 3.03^b^	395.98 ± 1.60^c^	389.40 ± 2.08^d^
**ALT (U/L)**	17.75 ± 0.55^a^	17.09 ± 0.38^a^	15.37 ± 0.41^b^	14.60 ± 0.34^b^
**GLU (mg/dL)**	59.97 ± 1.34^a^	50.63 ± 0.97^b^	46.25 ± 0.99^c^	41.70 ± 1.35^d^
**CHO (mg/dL)**	139.33 ± 1.52^a^	133.08 ± 0.87^b^	125.53 ± 1.29^c^	115.13 ± 1.49^d^
**TP (g/dL)**	3.46 ± 0.03^d^	3.51 ± 0.02^c^	3.65 ± 0.03^b^	3.78 ± 0.02^a^
**ALBUMN (g/dL)**	1.47 ± 0.02^a^	1.43 ± 0.01^b^	1.39 ± 0.02^c^	1.34 ± 0.02^d^
**GLOBLN (g/dL)**	2.06 ± 0.02^a^	2.02 ± 0.01^b^	1.89 ± 0.02^c^	1.80 ± 0.01^d^
**CRE (mg/dL)**	0.42 ± 0.00^a^	0.40 ± 0.00^b^	0.39 ± 0.01^c^	0.37 ± 0.01^d^

*Note*: There is a statistical difference between the data shown with different letters in the same row (*p* < 0.05).

Abbreviations: ALP, alkaline phosphatase; ALT, alanine aminotransferase; AST, aspartate aminotransferase; Cho, cholesterol; Glu, glucose; TP, total protein; TRG, triglyceride.

In contrast, GLU and CHO concentrations declined significantly in glutamine‐fed groups. The lowest values were recorded in fish receiving 2.0% glutamine (41.70 ± 1.35 mg/dL for GLU and 115.13 ± 1.49 mg/dL for CHOL), indicating a possible effect of glutamine on carbohydrate and lipid metabolic regulation.

Significant reductions were also noted in hepatic enzyme activities. ALP, AST and ALT levels were lowest in the G4 group (117.33 ± 0.12, 389.40 ± 2.08 and 14.60 ± 0.34 U/L, respectively), reflecting improved hepatic status and potentially reduced cellular stress in response to glutamine intake.

Additionally, ALB and GLO levels followed a decreasing trend in glutamine‐fed groups, whereas CRE levels were lowest in the G4 group (0.37 ± 0.01 mg/dL). These results indicate that dietary glutamine supplementation supports overall metabolic balance and enhances physiological homeostasis in female rainbow trout.

### 3.6 Oxidative Stress Response in Blood and Muscle Tissues of Rainbow Trout

The impact of dietary glutamine on oxidative stress biomarkers in blood and muscle tissues is presented in Table 6. In blood samples, TAS increased significantly with higher levels of glutamine, reaching 1.85 ± 0.02 mmol/L in the G4 group (*p* < 0.05). Concurrently, TOS also rose, with the highest concentration recorded in G4 (8.35 ± 0.36 µmol/L). The OSI increased in the G2 and G3 groups but remained stable in G4, indicating a balanced oxidative state at higher glutamine inclusion.

**TABLE 6:** Changes in oxidative stress profiles in blood and muscle tissues of glutamine‐fed rainbow trout.

	G1‐control	G2‐1.0%	G3‐1.5%	G4‐2.0%
**Blood oxidative stress parameters**				
TAS (mmol/L)	1.10 ± 0.07^c^	1.26 ± 0.03^b^	1.32 ± 0.03^b^	1.85 ± 0.02^a^
TOS (µmol/L)	2.41 ± 0.18^d^	4.38 ± 0.14^c^	6.03 ± 0.15^b^	8.35 ± 0.36^a^
OSI	0.22 ± 0.02^c^	0.35 ± 0.01^b^	0.46 ± 0.01^a^	0.45 ± 0.02^a^
MPO (U/L)	20.25 ± 0.96^d^	23.75 ± 2.22^c^	29.50 ± 1.29^b^	33.00 ± 1.41^a^
MDA (mmol/L)	19.03 ± 1.25^d^	25.25 ± 0.95^c^	27.75 ± 1.50^b^	32.75 ± 1.71^a^
**Muscle oxidative stress parameters**				
TAS (mmol/L)	9.15 ± 0.71^a^	6.89 ± 0.37^b^	9.63 ± 0.78^a^	9.15 ± 0.85^a^
TOS (µmol/L)	119.46 ± 56.20^a^	100.06 ± 30.54^b^	1114.78 ± 35.60^a^	941.11 ± 35.99^c^
OSI	12.83 ± 0.92^b^	14.68 ± 0.51^a^	11.62 ± 0.77^c^	10.33 ± 0.80^d^
MP	58.52 ± 1.86^b^	68.20 ± 1.23^a^	60.02 ± 3.33^b^	69.98 ± 1.40^a^
MPO (U/L)	1163.63 ± 44.85^b^	141.24 ± 46.49^a^	1343.06 ± 25.66^a^	1207.90 ± 69.51^b^
MDA (mmol/L)	210.39 ± 15.70^a^	168.64 ± 1.29^b^	162.29 ± 4.59^cd^	150.16 ± 7.31^d^

*Note*: Lowercase superscripts (a–c) indicate significant differences on the same colon within each experimental treatment group (*n* = 6, mean ± standard deviation).

Abbreviations: MDA, malondialdehyde; MP, microprotein; MPO, myeloperoxidase; OSI, oxidative stress index; TAS, total antioxidant status; TOS, total oxidant status.

Elevated levels of MPO and MDA in blood were also detected in the G4 group (33.00 ± 1.41 U/L and 32.75 ± 1.71 mmol/L, respectively), suggesting an upregulated antioxidant response.

In muscle tissue, TAS values were similar among groups, except for a significant reduction in G2. However, TOS levels declined in G4 (941.11 ± 35.99 µmol/L), reflecting reduced oxidative load. A corresponding decrease in OSI was observed with increasing glutamine inclusion, with the lowest value in G4 (10.33 ± 0.80). Muscle MDA concentrations followed a similar trend, showing the lowest level in the G4 group (150.16 ± 7.31 mmol/L), suggesting reduced lipid peroxidation and improved tissue oxidative stability.

Overall, these results indicate that dietary glutamine supplementation enhances antioxidant defence mechanisms and contributes to oxidative balance in both blood and muscle tissues of female rainbow trout.

## 4 Discussion

The results of this study indicate that dietary glutamine exerts multi‐level effects on the physiological status and quality characteristics of female rainbow trout. When considered collectively, the observed changes in growth performance, haematological parameters, oxidative stress markers and sensory traits suggest that glutamine functions beyond a conventional nutrient, potentially acting as a metabolic modulator.

Glutamine supplementation significantly enhanced key growth parameters, including FW, WG and SGR, particularly in the 1.5% and 2.0% inclusion groups. These results agree with findings in other species, such as Nile tilapia (Pereira et al. 2017) and Jian carp (Hu et al. 2015), in which glutamine improved protein utilization and feed efficiency. Mechanistically, these effects may involve increased enterocyte energy availability and enhanced amino acid transport (Li et al. 2020). Glutamine may stimulate analogous intracellular signalling pathways, such as the PI3K/AKT/mTOR pathway, which has been previously documented to be increased in glutamic acid‐fed largemouth bass (Jiang et al. 2024), hence facilitating muscle protein synthesis. Consistent with Jiang et al. (2024), who demonstrated that dietary glutamic acid enhanced growth and intestinal enzyme activity in largemouth bass, the current findings show that glutamine supplementation improves efficiency in feed of rainbow trout.

The observations of changes in PER and reduction in FCR suggest improved protein retention and metabolic efficiency. The increase in muscle crude protein content further supports this notion. Similar protein‐sparing effects have been linked to glutamine‐induced activation of the mTOR signalling pathway, which regulates protein synthesis in muscle tissues (Li et al. 2020). The reduction in lipid deposition may also reflect a shift toward anabolic lean tissue growth. In accordance with findings in Nile tilapia (Naena et al. 2024), this study's results demonstrate that dietary supplementation of 1.5%–2.0% of glutamine markedly enhanced growth performance and feed efficiency in female rainbow trout. This indicates that the beneficial impacts of glutamine on anabolic metabolism and nutrient utilization may be preserved among freshwater species.

The haematological improvements, elevated RBC count, Hb concentration and Hct values suggest better oxygen transport and possibly reduced oxidative burden. These parameters are frequently used as indicators of fish health status under intensive rearing, and their enhancement implies a potential stress‐mitigating effect of glutamine (Öz et al. 2024a, 2024b).

In terms of serum biochemistry, the reduction in GLU and CHOL levels in glutamine‐fed fish could indicate improved metabolic regulation. These shifts may be associated with increased insulin sensitivity or gluconeogenic control, as proposed in earlier studies on warmwater species (Karlsson et al. 2006; Zhang et al. 2024). The lowered activities of hepatic enzymes (AST, ALT and ALP) are also suggestive of reduced hepatic stress or tissue damage. Although histopathological data are lacking, such biochemical profiles are often interpreted as indicative of hepatoprotection (Ramos et al. 2020).

Glutamine‐fed groups exhibited improved antioxidant status, reflected by increased TAS and decreased MDA levels in both blood and muscle tissues. These effects may arise from glutamine's role in glutathione biosynthesis, as well as its indirect modulation of antioxidant enzyme activity (Coutinho et al. 2016). The reduction in OSI further confirms that glutamine contributes to maintaining redox homeostasis. Consistent with observations in crucian carp subjected to carbonate alkaline stress (Sun et al. 2024), the data indicate that glutamine supplementation in rainbow trout augments the antioxidant defence system, possibly through the activation of glutathione metabolism.

These elevated TOS and MPO levels likely represent an adaptive oxidative response rather than a detrimental effect. Controlled increases in oxidative mediators can reflect physiological immunometabolic adjustments, which are essential for maintaining cellular homeostasis under dietary and environmental stressors (Chamberlin and Ballantyne 1992).

The enhancement in sensory traits, particularly texture and flavour, is a practical outcome that adds commercial value. These improvements correlate with biochemical shifts, higher protein and lower fat content, and reduced lipid oxidation, which is known to impact sensory perception (Öz 2018). Although raw attributes remained unchanged, cooked fillets from glutamine‐fed groups received higher panel scores, supporting the utility of dietary glutamine in producing value‐added products. The previous research indicates that dietary content substantially influences sensory characteristics in fish, with functional feed additives being essential for enhancing product quality (Öz et al. 2017; Kocatepe et al. 2024; Öz 2018; Kaya Öztürk et al. 2019).

Improved muscle structure may also be attributed to reduced oxidative stress in muscle tissue, which can influence protein degradation and water‐holding capacity factors strongly linked to texture. However, the study did not assess fillet shelf life or consumer preference under real market conditions, which should be addressed in future trials.

Although glutamine supplementation improved several physiological and product quality parameters, its cost‐effectiveness in large‐scale trout production remains a critical consideration. Factors, such as ingredient price, feed conversion efficiency and market value of enhanced fillets, should be evaluated in future studies to determine their practical viability.

## 5 Conclusion

On the basis of the outcomes of this study, dietary glutamine appears to influence several performance‐ and quality‐related traits in female rainbow trout. The improvements in growth, protein retention, haematological stability and fillet texture indicate that glutamine has potential as a functional feed additive.

Nonetheless, some results, especially those related to oxidative stress, present complexity and require further clarification through mechanistic studies. The current findings may provide a useful basis for more targeted trials involving histology, gene expression or commercial‐scale testing.

In conclusion, although glutamine demonstrates promise in trout aquaculture, future work should consider optimal dosage, species‐specific responses and cost‐effectiveness before recommending routine inclusion in commercial feeds. Therefore, the inclusion of 1.5%–2.0% glutamine in grow‐out feeds can be considered a novel and promising nutritional strategy to enhance growth performance, muscle quality and antioxidant status in female rainbow trout, offering tangible commercial benefits for sustainable aquaculture.

## Author Contributions

**Mustafa Öz**: conceptualization, data curation, formal analysis, funding acquisition, investigation, methodology, project administration, resources, software, validation, visualization, review and editing writing – original draft preparation. **Burak Evren İnanan**: conceptualization, data curation, formal analysis, funding acquisition, investigation, methodology, project administration, resources, software, validation, visualization, review and editing writing – original draft preparation.

## Conflicts of Interest

The authors declare no conflicts of interest.

## Peer Review

The peer review history for this article is available at https://publons.com/publon/10.1002/vms3.70527

## Data Availability

The datasets are available from the corresponding author upon reasonable request.

## References

[vms370527-bib-0001] Alak, G. , F. B. Özgeriş , A. Ç. Yeltekin , et al. 2020. “Hematological and Hepatic Effects of Ulexite in Zebrafish.” Environmental Toxicology and Pharmacology 80: 103496. 32947019 10.1016/j.etap.2020.103496

[vms370527-bib-0002] AOAC . 1984. Official Methods of Analysis. 15th ed. Association of Official Analytical Chemist.

[vms370527-bib-0003] AOAC . 1990. Official Methods of Analysis of the Association of the Official Analysis Chemists. 15th ed. Association of Official Analytical Chemists.

[vms370527-bib-0004] Aussanasuwannakul, A. , P. B. Kenney , G. M. Weber , et al. 2011. “Effect of Sexual Maturation on Growth, Fillet Composition, and Texture of Female Rainbow Trout (*Oncorhynchus mykiss*) on a High Nutritional Plane.” Aquaculture 317, no. 1–4: 79–88.

[vms370527-bib-0005] Blaxhall, P. C. , and K. W. Daisley . 1973. “Routine Haematological Methods for Use With Fish Blood.” Journal of Fish Biology 5, no. 6: 771–781.

[vms370527-bib-0006] Bligh, E. C. , and W. J. Dyer . 1959. “A Rapid Method of Total Lipid Extraction and Purification.” Canadian Journal of Biochemistry and Physiology 37: 913–917. 10.1139/o59-099 13671378

[vms370527-bib-0007] Bonilla, A. C. , K. Sveinsdottir , and E. Martinsdottir . 2007. “Development of Quality Index Method (QIM) Scheme for Fresh Cod (*Gadus morhua*) Fillets and Application in Shelf Life Study.” Food Control 18, no. 4: 352–358.

[vms370527-bib-0008] Bradford, M. M. 1976. “A Rapid and Sensitive Method for the Quantitation of Microgram Quantities of Protein Utilizing the Principle of Protein‐Dye Binding.” Analytical Biochemistry 72, no. 1–2: 248–254. 942051 10.1016/0003-2697(76)90527-3

[vms370527-bib-0009] Bradley, J. R. , D. R. Johnson , and J. S. Pober . 1993. “Endothelial Activation by Hydrogen Peroxide. Selective Increases of Intercellular Adhesion Molecule‐1 and Major Histocompatibility Complex Class I.” American Journal of Pathology 142, no. 5: 1598–1609. 8098585 PMC1886909

[vms370527-bib-0010] Chamberlin, M. E. , and J. S. Ballantyne . 1992. “Glutamine Metabolism in Elasmobranch and Agnathan Muscle.” Journal of Experimental Zoology 264, no. 3: 267–272.

[vms370527-bib-0011] Company, R. , J. A. Calduch‐Giner , J. Perez‐ Sanchez , and S. Kaushik . 1999. “Protein Sparing Effect of Dietary in Common Dentex (*Dentex dentex*): A Comparative Study With Sea Bream (*Sparus aurata*) and Sea Bass (*Dicentrarchus labrax*).” Aquatic Living Resources 12: 23–30.

[vms370527-bib-0012] Coutinho, F. , C. Castro , E. Rufino‐Palomares , et al. 2016. “Dietary Glutamine Supplementation Effects on Amino Acid Metabolism, Intestinal Nutrient Absorption Capacity and Antioxidant Response of Gilthead Sea Bream (*Sparus aurata*) Juveniles.” Comparative Biochemistry and Physiology Part A: Molecular & Integrative Physiology 191: 9–17. 10.1016/j.cbpa.2015.09.012 26424608

[vms370527-bib-0013] Erel, O. 2004. “A Novel Automated Direct Measurement Method for Total Antioxidant Capacity Using a New Generation, More Stable ABTS Radical Cation.” Clinical Biochemistry 37, no. 4: 277–285. 15003729 10.1016/j.clinbiochem.2003.11.015

[vms370527-bib-0014] Erel, O. 2005. “A New Automated Colorimetric Method for Measuring Total Oxidant Status.” Clinical Biochemistry 38, no. 12: 1103–1111. 16214125 10.1016/j.clinbiochem.2005.08.008

[vms370527-bib-0015] FAO . 2025. The State of World Fisheries and Aquaculture 2022. FAO. 10.4060/cd4312en.

[vms370527-bib-0016] Han, Y. , S. Koshio , Z. Jiang , et al. 2014. “Interactive Effects of Dietary Taurine and Glutamine on Growth Performance, Blood Parameters and Oxidative Status of Japanese Flounder *Paralichthys olivaceus* .” Aquaculture 434: 348–354.

[vms370527-bib-0017] Harma, M. , M. Harma , and O. Erel . 2003. “Increased Oxidative Stress in Patients With Hydatidiform Mole.” Swiss Medical Weekly: Official Journal of the Swiss Society of Infectious Diseases, the Swiss Society of Internal Medicine, the Swiss Society of Pneumology 133: 563–566. 10.4414/smw.2003.10397 14691728

[vms370527-bib-0018] Hu, K. , J. X. Zhang , L. Feng , et al. 2015. “Effect of Dietary Glutamine on Growth Performance, Non‐Specific Immunity, Expression of Cytokine Genes, Phosphorylation of Target of Rapamycin (TOR), and Anti‐Oxidative System in Spleen and Head Kidney of Jian Carp (*Cyprinus carpio* var. Jian).” Fish Physiology and Biochemistry 41: 635–649. 25675866 10.1007/s10695-015-0034-0

[vms370527-bib-0019] Huang, Y. , M. Wang , J. Pan , et al. 2024. “Dietary Glutamine Supplementation Improves the Osmoregulatory Capacity and Reduces Oxidative Stress Induced by Hyperosmotic Stress in Nile Tilapia (*Oreochromis niloticus*).” Aquaculture Reports 37: 102267.

[vms370527-bib-0020] Jiang, F. , W. Huang , M. Zhou , et al. 2024. “Effects of Dietary L‐Glutamic Acid on the Growth Performance, Gene Expression Associated With Muscle Growth‐Related Gene Expression, and Intestinal Health of Juvenile Largemouth Bass (*Micropterus salmoides*).” Fishes 9, no. 8: 312.

[vms370527-bib-0021] Karlsson, A. , E. J. Eliason , L. T. Mydland , A. P. Farrell , and A. Kiessling . 2006. “Postprandial Changes in Plasma Free Amino Acid Levels Obtained Simultaneously From the Hepatic Portal Vein and the Dorsal Aorta in Rainbow Trout (*Oncorhynchus mykiss*).” Journal of Experimental Biology 209, no. 24: 4885–4894. 17142677 10.1242/jeb.02597

[vms370527-bib-0022] Kaya Öztürk, D. , B. Baki , R. Öztürk , S. Karayücel , and G. Uzun Gören . 2019. “Determination of Growth Performance, Meat Quality and Colour Attributes of Large Rainbow Trout (*Oncorhynchus mykiss*) in the Southern Black Sea Coasts of Turkey.” Aquaculture Research 50, no. 12: 3763–3775.

[vms370527-bib-0023] Kocatepe, D. , C. O. Altan , B. Corapci , I. Keskin , B. Kostekli , and H. Turan . 2024. “Correlation Between Texture Profile Analysis and Colour Characteristics of Fish: Case Study: Black Sea Large Rainbow Trout and Norwegian Salmon.” Bulletin of University of Agricultural Sciences and Veterinary Medicine Cluj‐Napoca. Food Science and Technology 86: 1–15.

[vms370527-bib-0024] Kosecik, M. , O. Erel , E. Sevinc , and S. Selek . 2005. “Increased Oxidative Stress in Children Exposed to Passive Smoking.” International Journal of Cardiology 100: 61–64. 15820286 10.1016/j.ijcard.2004.05.069

[vms370527-bib-0025] Li, X. , S. Zheng , and G. Wu . 2020. “Nutrition and Metabolism of Glutamate and Glutamine in Fish.” Amino Acids 52, no. 5: 671–691. 32405703 10.1007/s00726-020-02851-2

[vms370527-bib-0026] Manor, M. L. , B. M. Cleveland , P. Brett Kenney , J. Yao , and T. Leeds . 2015. “Differences in Growth, Fillet Quality, and Fatty Acid Metabolism‐Related Gene Expression Between Juvenile Male and Female Rainbow Trout.” Fish Physiology and Biochemistry 41: 533–547. 25673423 10.1007/s10695-015-0027-z

[vms370527-bib-0027] Naena, N. A. , E. T. Al‐Sokary , E. K. Abeer , S. Elbaz , and A. S. Kassab . 2024. “Effect of Glutamine on Growth Performance, Biochemical Parameters and Diseases Resistance in Cultured Nile Tilapia Fish.” Alexandria Journal of Veterinary Sciences 83: 114–126.

[vms370527-bib-0028] Öz, M. 2018. “Effects of Garlic (*Allium sativum*) Supplemented Fish Diet on Sensory, Chemical and Microbiological Properties of Rainbow Trout During Storage at −18°C.” LWT 92: 155–160.

[vms370527-bib-0029] Öz, M. 2025. “Effects of Boric Acid on Oxidative Stress Parameters, Growth Performance and Blood Parameters of Rainbow Trout (*Oncorhynchus mykiss)* .” Biological Trace Element Research 203: 1647–1655. 38913295 10.1007/s12011-024-04276-4 PMC11872762

[vms370527-bib-0030] Öz, M. , S. Dikel , M. Durmuş , and Y. Özoğul . 2017. “Effects of Black Cumin Oil (*Nigella sativa*) on Sensory, Chemical and Microbiological Properties of Rainbow Trout During 23 Days of Storage at 2±1°C.” Journal of Aquatic Food Product Technology 26, no. 6: 665–674.

[vms370527-bib-0031] Öz, M. , B. E. Inanan , T. Karasahin , and S. Dikel . 2024a. “Effects of Glutamine on Growth Performance, Nutrient Content, Fatty Acid Profile, and Blood Parameters of Rainbow Trout (*Oncorhynchus mykiss*).” Journal of Fish Biology 104, no. 4: 1213–1222. 38263635 10.1111/jfb.15666

[vms370527-bib-0032] Öz, M. , E. Üstüner , and F. Bölükbaş . 2024b. “Effects of Dietary Black Cumin (*Nigella sativa* L.) Oil on Growth Performance, Hemato‐Biochemical and Histopathology of Cypermethrin‐Intoxicated Nile Tilapia (*Oreochromis niloticus*).” Journal of the World Aquaculture Society 55, no. 1: 273–288. 10.1002/vms3.1449 PMC10998455 38581350

[vms370527-bib-0043] Öz, M. , B. E. Inanan , E. Üstüner , B. Karagoz , and S. Dikel . 2024c. “Effects of Dietary Garlic (*Allium sativum*) Oil on Growth Performance, Haemato‐Biochemical and Histopathology of Cypermethrin‐Intoxicated Nile Tilapia (*Oreochromis niloticus*).” Veterinary Medicine and Science 10, no. 3: e1449. 38581350 10.1002/vms3.1449 PMC10998455

[vms370527-bib-0033] Paulus, K. , R. Zacharias , L. Robinson , and H. Geidel . 1979. “Kritische Betrachtungen zur “Bewertenden Prüfung Mit Skale” Als Einem Wesentlichen Verfahren der Sensorischen Analyse.” LWT‐Food Science and Technology 12: 52–61.

[vms370527-bib-0034] Pereira, R. T. , P. V. Rosa , and D. M. Gatlin III . 2017. “Glutamine and Arginine in Diets for Nile Tilapia: Effects on Growth, Innate Immune Responses, Plasma Amino Acid Profiles and Whole‐Body Composition.” Aquaculture 473: 135–144.

[vms370527-bib-0035] Ramos, A. P. S. , J. R. Luz , J. F. B. Melo , and L. G. T. Braga . 2020. “Glutamine Use in Feeding Juvenile Pirarucu, Arapaima Gigas (Schinz, 1822).” Arquivo Brasileiro De Medicina Veterinária e Zootecnia 72: 1789–1796.

[vms370527-bib-0036] Santinha, P. J. M. , F. Medale , G. Corazze , and E. F. S. Gomes . 1999. “Effects of the Dietary Protein: Lipid Ratio on Growth and Nutrient Utilization in Gilthead Seabream *Sparus aurata* L.” Aquaculture Nutrition 5: 147–156.

[vms370527-bib-0037] Skalli, A. , M. C. Hidalgo , E. Abellán , M. Arizcun , and G. Cardenete . 2004. “Effects of the Dietary Protein/Lipid Ratio on Growth and Nutrient Utilization in Common Dentex (*Dentex dentex* L.) at Different Growth Stages.” Aquaculture 235, no. 1–4: 1–11.

[vms370527-bib-0038] Sun, Y. , C. Geng , W. Liu , Y. Liu , L. Ding , and P. Wang . 2024. “Investigating the Impact of Disrupting the Glutamine Metabolism Pathway on Ammonia Excretion in Crucian Carp (*Carassius auratus*) Under Carbonate Alkaline Stress Using Metabolomics Techniques.” Antioxidants 13, no. 2: 170. 38397768 10.3390/antiox13020170 PMC10885916

[vms370527-bib-0039] Wicks, B. J. , and D. J. Randall . 2002. “The Effect of Sub‐Lethal Ammonia Exposure on Fed and Unfed Rainbow Trout: The Role of Glutamine in Regulation of Ammonia.” Comparative Biochemistry and Physiology Part A: Molecular & Integrative Physiology 132, no. 2: 275–285. 10.1016/s1095-6433(02)00034-x 12020644

[vms370527-bib-0040] Yamashita, M. , and S. Konagaya . 1990. “High Activities of Cathepsins B, D, H, and L in the White Muscle of Chum Salmon in Spawning Migration.” Comparative Biochemistry and Physiology. B, Comparative Biochemistry 95, no. 1: 149–152. 2331869 10.1016/0305-0491(90)90262-r

[vms370527-bib-0041] Yumru, M. , H. A. Savas , A. Kalenderoglu , M. Bulut , H. Celik , and O. Erel . 2009. “Oxidative Imbalance in Bipolar Disorder Subtypes: A Comparative Study.” Progress in Neuro‐Psychopharmacology & Biological Psychiatry 33, no. 6: 1070–1074. 19527764 10.1016/j.pnpbp.2009.06.005

[vms370527-bib-0042] Zhang, B. , Q. Yang , N. Liu , Q. Zhong , and Z. Sun . 2024. “The Effects of Glutamine Supplementation on Liver Inflammatory Response and Protein Metabolism in Muscle of Lipopolysaccharide‐Challenged Broilers.” Animals 14, no. 3: 480. 38338123 10.3390/ani14030480 PMC10854980

